# The curcumin analogue hydrazinocurcumin exhibits potent suppressive activity on carcinogenicity of breast cancer cells via STAT3 inhibition

**DOI:** 10.3892/ijo.2011.1298

**Published:** 2011-12-14

**Authors:** XIAOFEI WANG, YAN ZHANG, XIWEN ZHANG, WENXIA TIAN, WENLI FENG, TINGMEI CHEN

**Affiliations:** Key Laboratory of Diagnostic Medicine Designated by the Ministry of Education, Chongqing Medical University, Chongqing 400016, P.R. China

**Keywords:** curcumin analogue, signal transducer and activator of transcription 3, breast cancer

## Abstract

Curcumin, the active component of turmeric, has been shown to protect against carcinogenesis and prevent tumor development in cancer. In our study, we tested the efficacy of a synthetic curcumin analogue, known as hydrazinocurcumin (HC), in breast cancer cells. The results demonstrated that compared to curcumin, HC was more effective in inhibiting STAT3 phosphorylation and downregulation of an array of STAT3 downstream targets which contributed to suppression of cell proliferation, loss of colony formation, depression of cell migration and invasion as well as induction of cell apoptosis. It was concluded that HC is a potent agent in the inhibition of STAT3 with more favorable pharmacological activity than curcumin, and HC may have translational potential as an effective cancer therapeutic or preventive agent for human breast carcinoma.

## Introduction

Many anticancer therapies currently in use are inadequate not only in terms of their therapeutic efficacy but also because they have undesirable side effects. On the other hand, certain dietary constituents known as phytochemicals have been shown to have significant anticancer efficacy (1) while causing minimal deleterious side effects.

**Figure 1:**
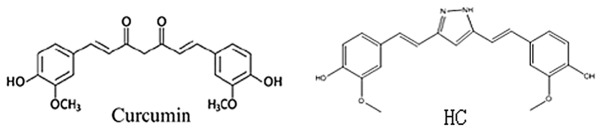
Chemical structures of curcumin and HC.

Curcumin, the active component of turmeric and a polyphenolic compound, is one of the most widely characterized phytochemicals. It has been a part of therapeutic preparations for centuries due to its wide spectrum of beneficial activities and its safety in relatively large dose (2). Evidence has been shown that curcumin inhibits the initiation, progression and continued survival of cancers cells (3). On basis of its numerous anti-carcinogenic properties, curcumin has already been the subject of several clinical trials for use as a treatment in human cancers. However, the low bioavailability prevents its use in chemotherapeutic application, and one potential means of circumventing this problem has been the creation of synthetic curcumin analogues. Hydrazinocurcumin (HC) (Fig. 1), a synthetic analogue of curcumin, was obtained as pale yellow gum which was analyzed for C_21_H_20_N_2_O_4_ by HRMS, thus 13° of unsaturation (4). Compared with curcumin, HC has greatly improved water solubility and stability, and has high cell permeability, or improved bioavailability with more favorable pharmacological activity (5). In our study, we compared the effects of HC and curcumin on carcinogenicity of breast cancers, and demonstrated for the first time that HC was more effective than curcumin in suppressing cell proliferation, colony formation, cell migration, invasion, and induction apoptosis in human breast cancer cells (MDA-MB-231, MCF-7), along with inhibition of STAT3 phosphorylation (Tyr705) and downregulation of STAT3 downstream targets. Therefore, we concluded that HC is substantially more effective than curcumin *in vitro*.

## Materials and methods

### Cell lines and reagents

The human breast cancer cell lines MDA-MB-231 and MCF-7 were obtained from Institute of Cell Research, Chinese Academy of Sciences. These cell lines were grown at 37˚C in Dulbecco's modified Eagle's medium (DMEM) with 10% fetal bovine serum (FBS, Sijiqing, China) in a humidified 5% CO_2_ incubator. All cells were washed three times in pH 7.4 PBS before harvesting for different experiments. Curcumin and HC were synthesized and provided kindly by Dr Yanmei Zhang at Carlifornia University of San Diego, the purity was >98%.

### MTT cell viability assay

Breast cancer cell lines (MDA-MB-231, MCF-7) were seeded in 96-well plates at a density of 3,000 cells per well. Different concentrations of curcumin (2.5–40 μM) or hydrazinocurcumin (0.5–5 μM) were added in triplicate to the plates in the presence of 10% FBS. The cells were incubated at 37˚C for 72 h. Then 25 μl MTT (Sigma) was added to each sample. After 3.5 h, 100 μl DMSO (Sigma) was added to each well. The absorbance was read at 490 nm. The viability of the untreated cells was arbitrarily set at 100% and compared with the viability of curcumim, hydrazinocurcumin-treated cells. IC_50_ was determined using SPSS 16.0 software.

### Colony formation assay

A base 0.6% agar gel with 10% FBS in DMEM was prepared and added to the well of a 6-well culture dish. Breast cancer cells were plated at a density of 3,000 cells per well on top of the base agar for anchorage-independent growth analysis in 0.4% agar gel with 10% FBS in DMEM supplemented with curcumin, hydrazinocurcumin or DMSO. The cells were maintained at 37˚C and allowed to grow for 2 weeks. The colonies were stained using MTT dye (100 μl per well). Pictures of the colonies were taken using a Leica MZ 16FA inverted microscope (Leica Microsystems, Bannockburn, IL) with a 7.4 Slider Camera (Diagnostic Instruments, Inc., Sterling Heights, MI). The colonies were scored by counting with an inverted microscope, and numbers were normalized as a percentage of colonies formed in DMSO treatment.

### Cell cycle analysis

Cell cycle phase was determined by fluorescence-activated cell sorting analysis. MDA-MB-231 and MCF-7 cells were inoculated into 6-well plates at a concentration of 5×10^5^ per well, exposed to curcumin and HC at a concentration of 10 μM, cultured for 24 and 48 h, collected, and sorted using flow cytometry (Bekman Coulter, USA) as described previously (6).

### Apoptosis rate analysis

MDA-MB-231 and MCF-7 cells were grown for 24 h in a 6-mm plate and then treated with the 10 μM of curcumin and HC for 24 and 48 h. Cells were washed by PBS 3 times, and then digested with 0.25% tryptan-EDTA. After centrifugation, cells were resuspended in 0.5 ml PBS. Cells were then stained with Annexin V and propidium iodide (PI) in the presence of 100 mg/ml RNAse and 0.1% Triton X-100 for 30 min at 37˚C. Flow cytometric (Bekman Coulter) analysis was performed using a fluorescence-activated cell sorter.

### Wound healing/cell migration assay

Breast cancer cells (3×10^5^ per well) were seeded in a 6-well plate. Approximately 24 h later, when the cells were 100% confluent, the monolayer was scratched using a 1-ml pipette tip and washed once to remove nonadherent cells. New medium in the presence of 10% FBS containing curcumin, hydrazinocurcumin or DMSO was added. The treatments were removed after 4 h, the fresh medium was added. After an additional 20 h without treatment, the cells were observed under a microscope. When the wound in the control was closed, the inhibition of migration was assessed by using the ImageJ software, available from the National Institutes of Health Web site (http://rsb.info.nih.gov/ij). The percent of wound healed was calculated using the formula: 100−[(final area/initial area) × 100%].

### Invasion (transwell) assay

To evaluate the effects of curcumin and HC on the invasion ability of MDA-MB-231 and MCF-7 cells, the cells were harvested by trypsinization after treatment for 24 h with curcumin and HC at doses of 10, 20 μmol/l. Cells were then seeded at a density of 50,000 cells in 0.2 ml in upper compartment. In the lower compartment 0.5 ml of medium was supplemented with 15% FBS. After incubation for 24 h, the upper cell layer was removed with a cotton swab, and the cells on the lower side were fixed with 4% formaldehyde solution. Subsequently, cells were stained with crystal viola and counted under a microscope.

### Western blot analysis

Breast cancer cell lines (MDA-MB-231, MCF-7) were treated with curcumin (10 or 20 μM, hydrazinocurcumin (10 or 20 μM) or DMSO for 24 h. For Western blot analysis, the protein from cancer cell lysates were subjected to SDS-PAGE and transferred to PVDF membrane. Blots were probed with phosphospecific STAT3 (Tyr 705) antibody (Cell Signaling Technologies), with STAT3 antibody (BD), with c-Myc, Mcl-1, Bcl-xl, survivin, MMP-9, MMP-2 antibodies (Bioword), with cyclin D1, VEGF, Bcl-2, BAX, β-actin antibodies (Santa Cruz Biotechnology), with PARP, caspase 9 antibodies (Beyotime). Membranes were analyzed using enhanced chemiluminescence (ECL) detection system (Viagene).

## Results

### HC and curcumin inhibit cell viability in human breast cancer cells

A dose-dependent inhibition in tumor cell proliferation/viability was observed after 72 h of treatment, and IC_50_ values were calculated for curcumin and HC (Table I). The results showed that compared with curcumin, HC was substantially more potent in inhibiting cell viability in both cell lines.

### Anchorage-independent growth and cell viability

**Figure 2:**
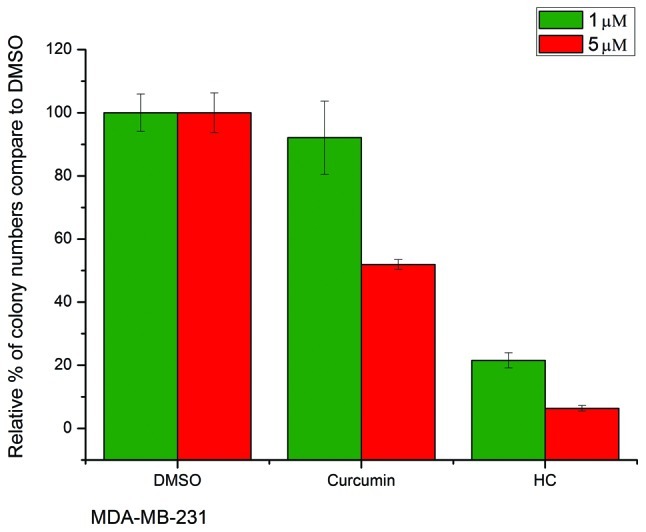

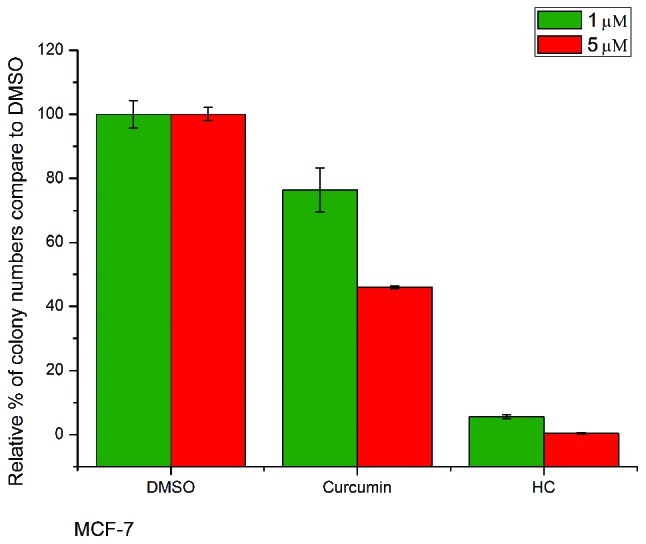
Colony formation assay. Colony formation of breast cancer cells in soft agar was inhibited by curcumin and HC. The potency of curcumin and HC were assessed in an anchorage-independent environment through a colony formation assay. Treatment with 1, 5 μmol/l curcumin and HC for 2 weeks greatly decreased the ability of MDA-MB-231 (A), MCF-7 (B) cells to form colonies in comparison to DMSO control (p<0.05).

The ability of transformed cells to grow and proliferate in the absence of substratum attachment is one of the hallmarks of malignancy and vitally important in the formation of the tumor (7). It was showed that treatment with curcumin and HC led to decreased colony formation in soft agar in both two cell lines (Fig. 2). Compared with the DMSO control samples, a 5 μmol/l concentration of curcumin elicited a decrease of ~50% in colony formation, while equal concentration of HC showed ~95% reduction in colony number.

### HC and curcumin arrest the cell cycle in human breast cancer cells

**Figure 3:**
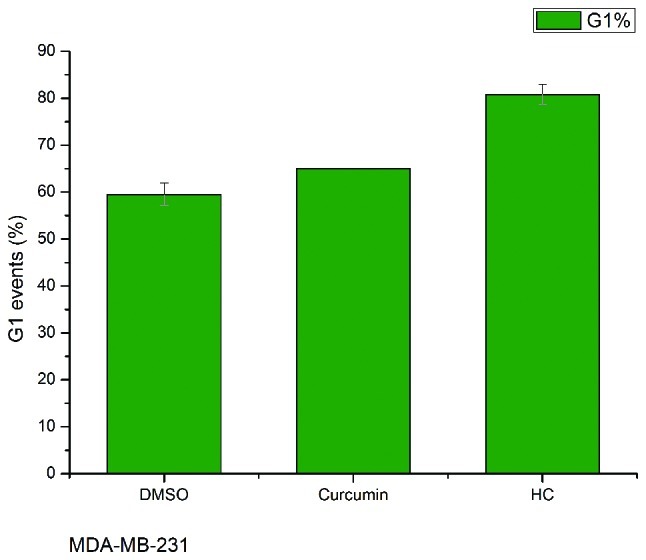

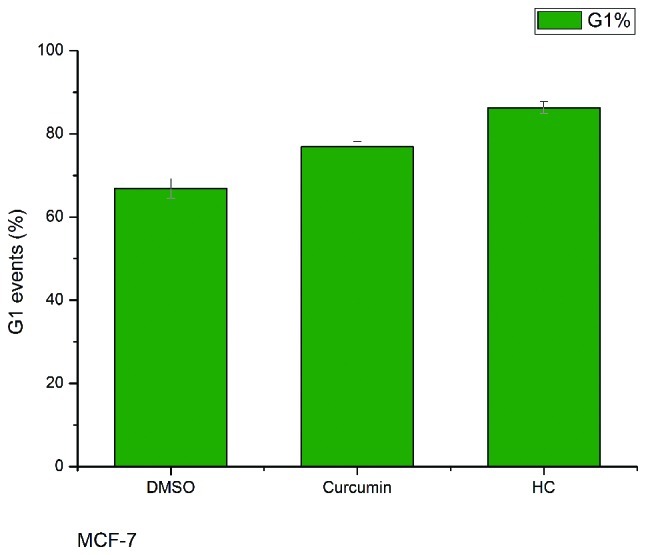
Cell cycle analysis. Treatment with 10 μM, curcumin and HC for 48 h had an effect on cell cycle progression of MDA-MB-231 and MCF-7 breast cancer cells as determined by FCM. Increased percentage of cells in G_1_ phase was observed in MDA-MB-231 (A), MCF-7 (B).

As shown (Fig. 3), HC and curcumin treatment at a concentration of 10 μM significantly increased the cell number in the G_1_ phase in MDA-MB-231 and MCF-7 (p<0.05 compared with control), and HC was more potent than curcumin in arresting the cells cycle in G_1_ phase.

### HC and curcumin induce cell apoptosis in human breast cancer cells

**Figure 4:**
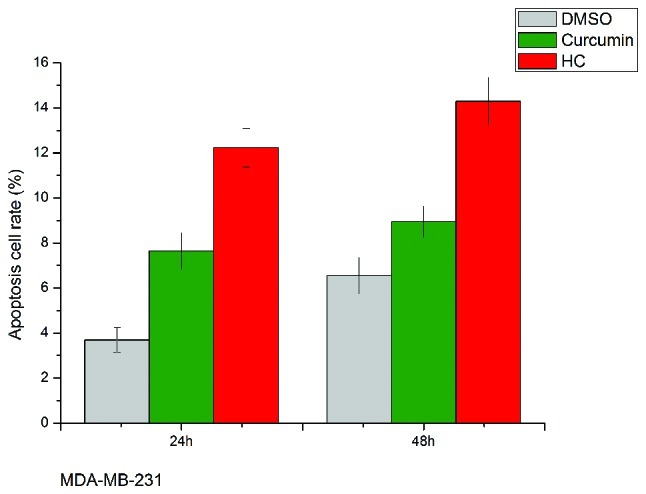

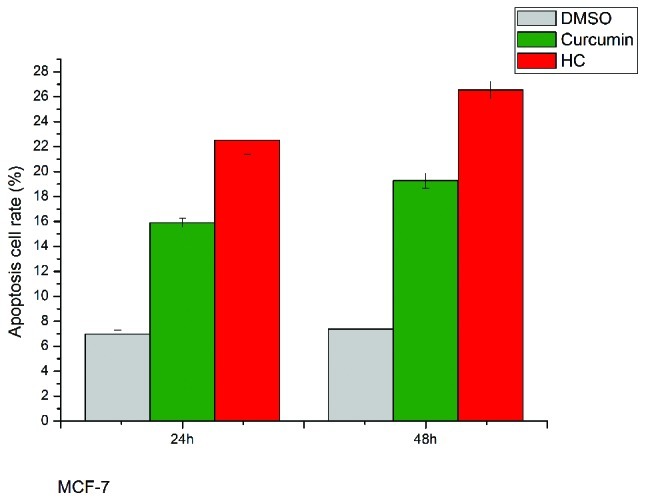
Apoptosis rate analysis. Induction of breast cancer cell apoptosis by curcumin and HC. Treatment with 10 M curcumin and HC for 24 and 48 h induced apoptosis in MDA-MB-231 (A), MCF-7 (B) cells as determined by FCM.

At a sufficiently high dose, curcumin induces apoptosis of many cancer cells including breast cancer cells. We assessed the effect of HC and curcumin on the induction of apoptosis in MDA-MB-231 and MCF-7 cells by FCM. The results (Fig. 4) showed that HC dose-dependently increased cells apoptotic rate after 48-h treatment; HC at 10 μmol/l significantly induced cells apoptosis (14% in MDA-MB-231 cells and 26% in MCF-7 cells), and at the same concentration, curcumin only induced 9 and 20% cells apoptosis in MDA-MB-231 and MCF-7 cells, respectively. In addition, PARP and caspase 9 are key effector molecules in the apoptosis pathway, and the enhancement of the level of the two molecules were also observed within 24 h in both MDA-MB-231 and MCF-7 cells treated with 10–20 μM curcumin and HC.

### HC and curcumin inhibit cell migration in human breast cancer cells

**Figure 5:**
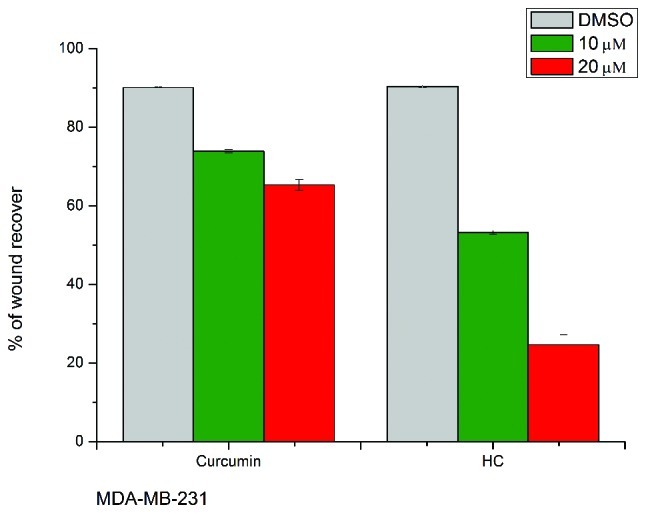

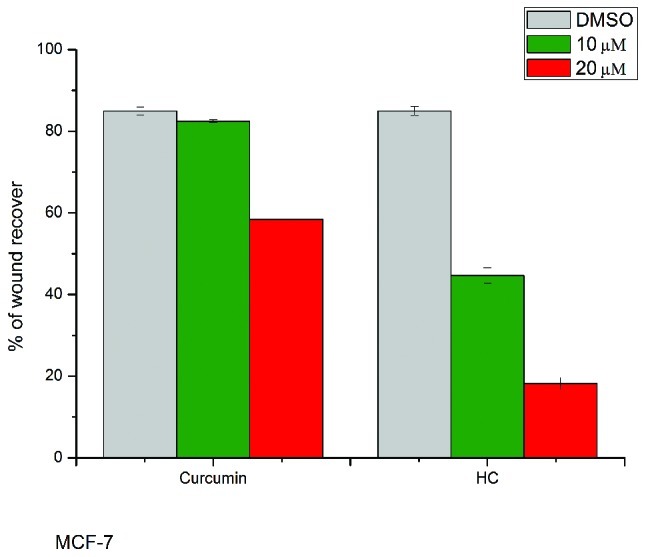

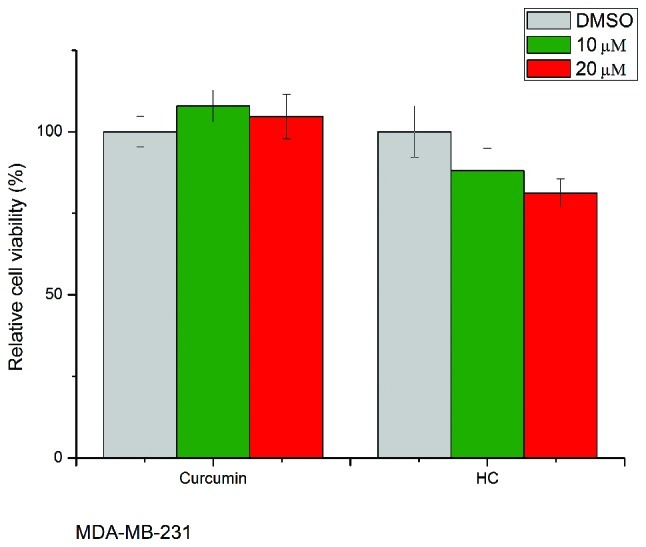

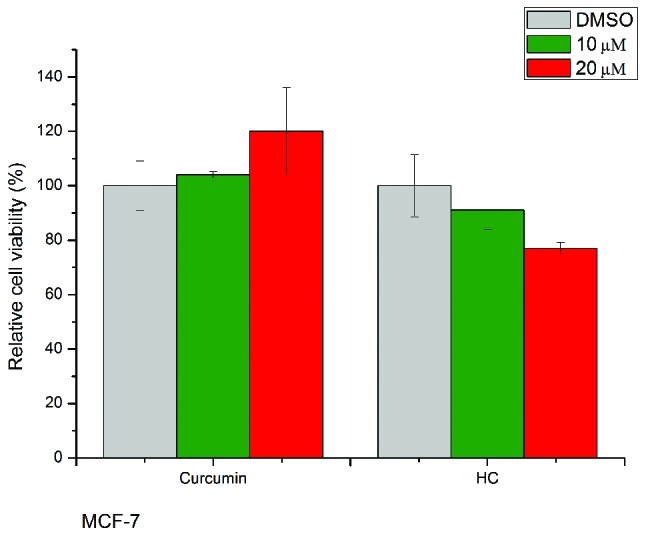
Wound healing assay. Curcumin and HC had an impact on breast cancer cells migration. Migration of breast cancer cells into a scratch wound was impeded after 4 h of treatment with 10 and 20 μM curcumin and HC. (A) MDA-MB-231, (B) MCF-7. MTT assays of MDA-MB-231 (C) and MCF-7 (D) cells revealed that the dosages of these agents used in the migration assay had minimal impact on viability over 4 h of drug treatment and an additional 20 h without treatment.

Cell migration is necessary in many physiological processes, such as wound healing and tumor metastasis. To investigate the effect of HC on cell migration, a wound healing assay was carried out with MDA-MB-231 and MCF-7 breast cancer cells. After the creation of a wound, cells were treated with various concentrations of HC and curcumin. Treatment was removed after 4 h and cells were returned to standard media in an attempt to minimize any cytotoxic effects that could potentially confound our observations. Following 20 h of further incubation, the areas of the wounds were measured using ImageJ software. Treatment with HC at a concentration of 10 μM or higher caused a significant decrease in cell migration (p<0.05) (Fig. 5). Because MTT assay revealed that the dosages and time points used in migration assay had minimal impact on cell viability (Fig. 5), the ability of HC and curcumin to inhibit cell migration might not be due to its ability to inhibit cell proliferation.

### HC and curcumin suppress cell invasion in human breast cancer cells

**Figure 6:**
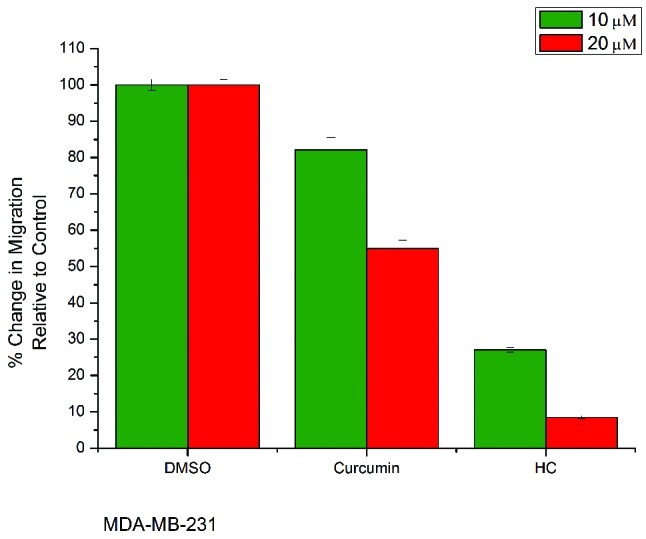

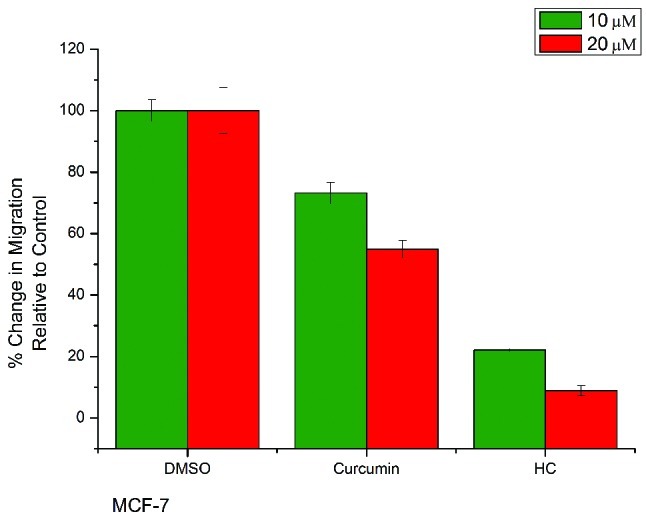
Invasion assay. Curcumin and HC inhibit invasion of breast cancer cells. Treatment of MDA-MB-231 (A) and MCF-7 (B) by 10 and 20 μM curcumin and HC for 24 h significantly reduced cell invasive behavior as assessed by transwell assay. Microscopic images reflect representative crystal violet staining of migrated MDA-MB231 cells 24 h after treatment.

To determine the role of curcumin and HC in cell invasion, MDA-MB-231 and MCF-7 cells were treated with indicated concentrations of curcumin and HC for 24 h. Both HC and curcumin significantly inhibited the invasion of two cell lines (Fig. 6). Interestingly, compared with the DMSO control, the invasion of MDA-MB-231 and MCF-7 cells was inhibited by ~90% at the concentration of 20 μM of HC, and equal administration of curcumin showed ~50% reduction in cell number.

### HC and curcumin inhibit the expression of STAT3 protein and its downstream targets in human breast cancer cells

**Figure 7:**
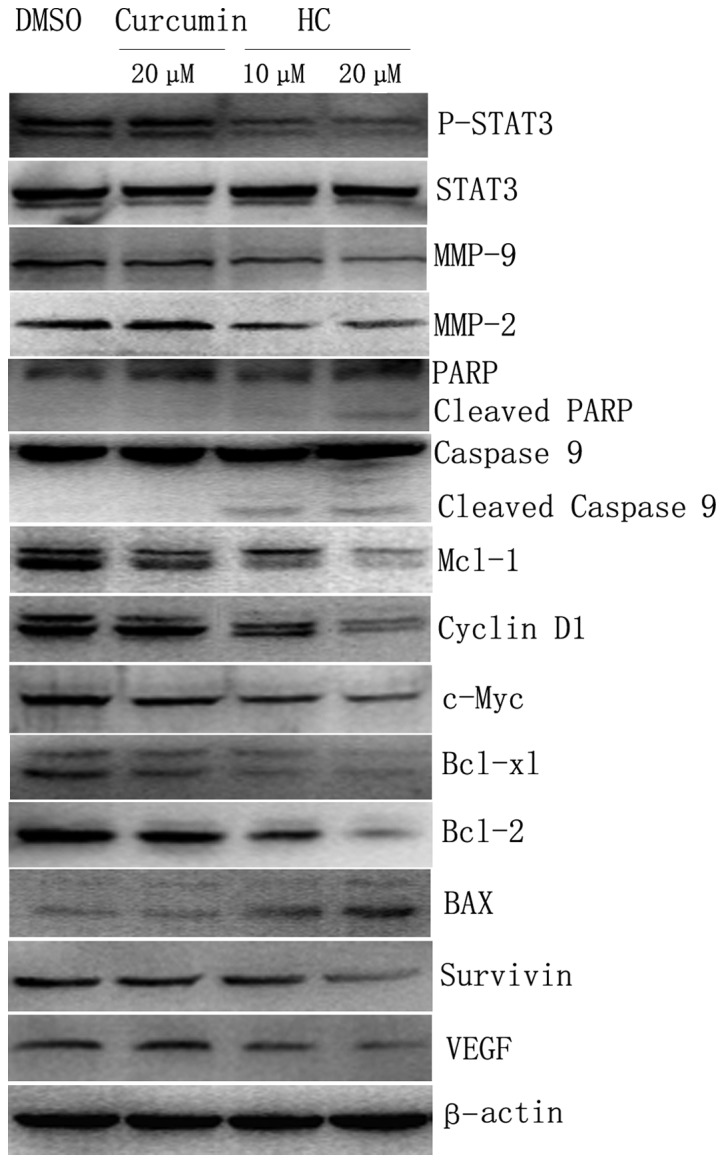

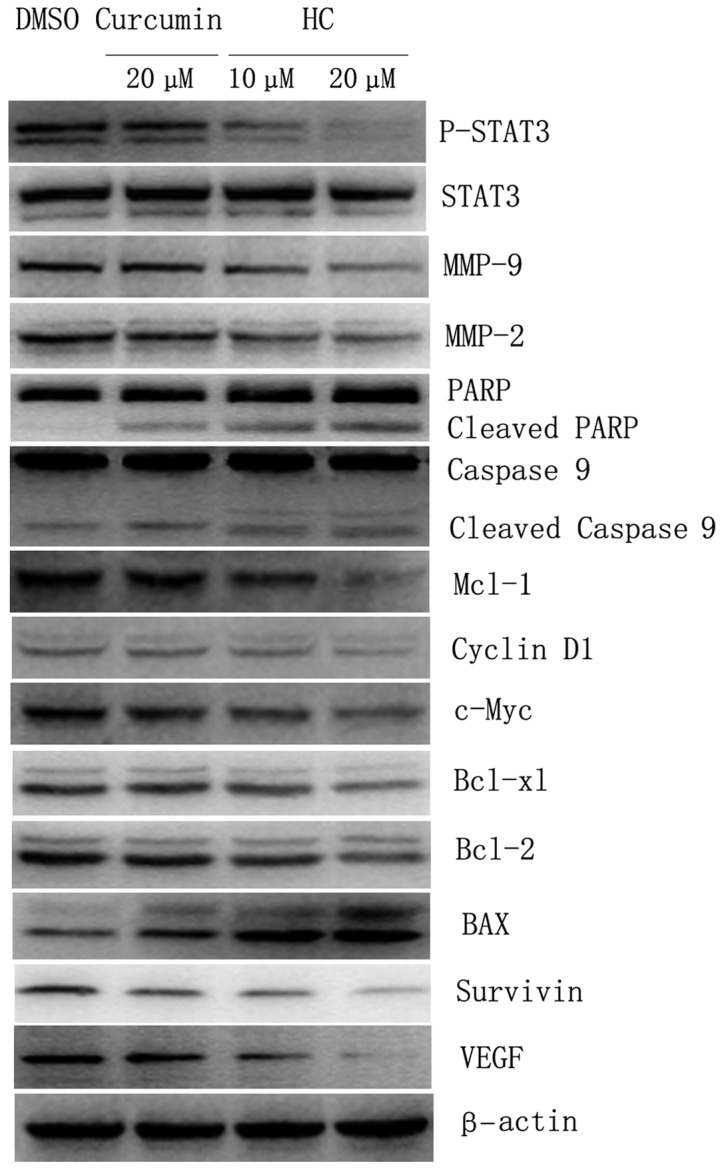
Expression of STAT3 and its downstream targets by Western blot analysis. Western blot analysis of cells treated with curcumin and HC. Both cancer cell lines express constitutively active STAT3. (A) MDA-MB-231, (B) MCF-7, exhibited decreased expression of STAT3 phosphorylation after treatment with 10 and 20 μM/l curcumin and HC for 24 h. Downstream targets of STAT3, including MMP-9, MMP-2, Mcl-1, cyclin D1, c-Myc, Bcl-xl, Bcl-2, survivin, and VEGF were also inhibited. Cell apoptosis is indicated by the induction of cleaved PARP, cleaved caspase 9 and BAX.

As previously mentioned, STAT3 binding to the promoters of the target genes induces the transcription of several proteins involved in carcinogenicity of cancer cells. As seen in Western blot assay (Fig. 7), HC was more potent than curcumin in inhibiting the expression of STAT3 protein at the same concentration (10–20 μM) in MDA-MB-231 and MCF-7 cells. To further analyze the expression of the downstream targets of STAT3, Western blotting was run for MMP-9, MMP-2, Mcl-1, cyclin D1, c-myc, Bcl-xl, Bcl-2, survivin and VEGF, and it found that treatment with HC resulted in more inhibition of the expression of these targets of STAT3 than curcumin.

## Discussion

The dysregulation of multiple oncogenic pathways is common among many cancers, including breast cancer cells. Being a phytochemical, anticancer efficacy of curcumin is mediated through regulating various transcription factors, growth factors, inflammatory cytokines, protein kinases, and other enzymes (8). Persistent activation of signal transduction and activators of transcription-3 (STAT3) is found with high frequency in a wide range of human cancer cell lines and tissues and implicated in stimulating cell proliferation, promoting angiogenesis, invasion and migration, mediating immune evasion, and conferring increased resistance to apoptosis (9–14). As one of the most important curcumin molecular targets, STAT3 can also be down-regulated effectively by curcumin (15,16).

STAT3 activation occurs when the tyrosine 705 (Tyr705) residue is phosphorylated, and then leads to dimerization and translocation from the cytoplasm to the nucleus (17–19). In the nucleus, STAT3 binding to target genes induces the transcription (20,21). Stat3 drives malignant progression through the deregulation of key proteins, including cell survival proteins such as Bcl-XL, Bcl-2, Mcl-1 and survivin (10,22–26), cell growth proteins such as cyclin D1/D2 and c-myc (9,27–31), inducers of angiogenesis such as vascular endothelial growth factor (VEGF) (32,33), and stimulators of invasion and metastasis such as MMP-2, MMP-9 (34–37). The crucial role of STAT3 in cancer progression and tumorigenesis suggests that it is a promising molecular target for cancer treatment.

Although curcumin has been shown to be a safe and effective inhibitor of STAT3, its low bioavailability prevents its chemotherapeutic application. In this study, we characterized the biologic activity of HC, a curcumin analog with improved water solubility, higher cell permeability and more favorable pharmacological activity; we evaluated for the first time the inhibitory efficacy of HC and curcumin in two breast cancer cells, MDA-MB-231 and MCF-7, which present constitutive activation of STAT3.

By both annexin V/PI staining and detection of cleavage of caspase 9, and PARP, it showed that compared with curcumin, HC increased the efficacy of induction of apoptosis of breast cancer cells. Accordingly, Bcl-2, Bcl-xl and Mcl-1, which are well-known apoptosis inhibitors and downstream target genes of STAT3, decreased after treatment with HC and curcumin. Bcl-xl and Bcl-2 are the anti-apoptotic protein within the Bcl-2 family that inhibits apoptosis by binding proapoptotic proteins and preventing cytochrome c release (38–40). Mcl-1 also represents a survival factor for human cancer cells (23,41). In addition, survivin is one of the key regulators of both cell cycle and apoptosis (42), and was shown in our study to be down-expressed.

The results of FCM also showed that treatment with curcumin and HC resulted in G_1_ arrest of cancer cells, and the decreased expression of cyclin D1 and c-Myc by Western blotting might account for proliferation inhibition and cell cycle arrest of the treated cells. Previous studies confirmed that Stat3 made an essential contribution to the regulation of cyclin D1 and c-Myc in v-Src transformation (43). Overexpression of cyclin D1 drives oncogenesis to regulate cell cycle progression (44). The c-Myc protooncogene is over-expressed in Burkett's lymphoma, and in other carcinomas such as breast cancer and colon cancer where it contributes to increased cellular proliferation and inhibition of differentiation (45–47).

The wound healing assay and transwell assay demonstrated that HC was more potent than curcumin in reducing the ability of migration and invasion of MDA-MB-231 and MCF-7 cells, and the level of MMP-2 and MMP-9 were also decreased. Constructive expression of STAT3 can up-regulate the expression of MMP-2 and MMP-9, which are able to efficiently degrade the extracellular matrix and basement membrane, promoting invasion and metastasis of cancer cells (34). Recent studies have found that the activity of STAT3 is closely related to the expression of MMP-9 in human breast cancer, and the activity of MMP-9, in epithelial cells of breast cancer, was significantly increased due to the sustained activation of the transformation of the plasmid by STAT3 (35). Thus, STAT3 may promote the progression of breast cancer through the regulation on MMP-9. Other studies have confirmed that MMP-2 might be the downstream target gene of STAT3; when it is translocated to the nucleus, phosphorylated STAT3 can bind to the promoter of MMP-2 to regulate MMP-2 expression (36,37).

In conclusion, our results showed that compared with curcumin, HC was more effective in inhibiting STAT3 phosphorylation and down-regulating an array of STAT3 downstream targets which contributed to the suppression of cell proliferation, loss of colony formation, depressing cell migration and invasion as well as induction of cell apoptosis. It was concluded that HC is a potent agent in inhibiting the phosphorylation of STAT3 with a more favorable pharmacological activity than curcumin, and HC might have translational potential as an effective drug or preventive agent for human breast carcinoma.

## Figures and Tables

**Table I:** IC_50_ (μM) of HC and curcumin in breast cancer cells.

Cell line	Curcumin	HC
MDA-MB-231	26.9	3.37
MCF-7	21.22	2.56

Cancer cells were treated for 72 h, and cell viability was analyzed by MTT assays. IC_50_ value (μM) was subsequently determined for each cell line.
